# Parental Responses to Online Sexual Grooming Events Experienced by Their Teenage Children

**DOI:** 10.3390/ejihpe14050086

**Published:** 2024-05-07

**Authors:** Michal Dolev-Cohen, Tamar Yosef, Michala Meiselles

**Affiliations:** 1 Oranim Academic College of Education, Kiryat Tiv’on 36006, Israel; 2 Law School, University of Derby, Derby DE21 1DZ, UK; m.meiselles@derby.ac.uk

**Keywords:** online sexual grooming, adolescents, parents, intervention programs

## Abstract

Online sexual grooming (a manipulative process in which the perpetrator locates a young person and creates an abusive relationship with the child that involves sexual exploitation) poses significant challenges to parents. This study examined how parents of adolescent victims of online sexual grooming experienced guiding their children through the event. This qualitative study, conducted in Israel, was based on semi-structured in-depth interviews with 15 parents who guided their adolescents who had been subjected to online sexual grooming. Results indicate that the parents reported a spectrum of emotions, from insecurity and guilt to a sense of control and satisfaction in managing the situation. Also, the reluctance of some parents to engage with the education system indicates potential trust issues. The study demonstrates the urgent need for targeted interventions to equip parents and educational professionals with the necessary knowledge for prevention and effective response to online sexual grooming. Implications for future research, policy, and practice are discussed.

## 1. Introduction

Adolescence is a time of exploration and development, characteristics that can make adolescents vulnerable to online risks like sexual grooming (a manipulative process in which the perpetrator locates a young person and creates an abusive relationship that involves sexual exploitation). Adolescence is a period characterized by many changes related to the development of sexual maturity, relationships with parents and peers, and the development of cognitive and emotional abilities [1]. Alongside these changes, this period is characterized by increased involvement in risk-taking behaviors [2]. According to the research literature, taking a risk means participating in risky behavior that involves a chance of loss [3]. It is part of a decision-making process that takes into account the expected reward from engaging in the behavior and the risk involved. This process is different for adolescents than for adults, as adolescents tend to prefer high short-term benefits even if the risk is high, a pattern that contributes to impulsive decision-making that may lead to losses in the long term [4]. Furthermore, going along with suggested sexual activity is simpler for adolescents, given their natural curiosity about sex, tendency to search for thrills, impulsiveness, and desire to have romantic relationships [5]. These characteristics serve as fertile ground for many potential online risks, including sexual exploitation [6,7].

### 1.1. Sexual Grooming

Some describe grooming as a premeditated behavior intended to gain the trust of a child and the first step in a process leading to future engagement in sexual conduct [8]. This process can last days, months, or even years, and is typically part of a broader strategy involving an array of techniques [9,10]. Ezioni highlighted the manipulative and occasionally prolonged nature of the process, which manifests itself in the steps taken by the perpetrator to groom the minors and make them believe their intentions are genuine and in their best interest [9]. Grooming does not always entail a face-to-face meeting, explicit conversations of a sexual nature, or online enactment of fantasies, but it typically involves the intention to exploit a child sexually. For this reason, O’Connell argued that online grooming is a subset of cybersexploitation [10].

Traditional grooming often involves persons known to the minor but online grooming is far broader. The perpetrator is often unknown to the minor and may even be in a different country. The Internet, social media, and other virtual platforms provide ample opportunity for perpetrators looking to engage anonymously in online grooming. Platforms that allow anonymous activity may create favorable conditions for the development of manipulative relationships between adults and young people, potentially leading to online sexual exploitation and abuse [11]. The way adolescents communicate online, where conversations with strangers are acceptable, whether in online games or textual exchanges, also facilitates interactions that can become dangerous [12,13]. Furthermore, the online environment often allows perpetrators to hide their true identity by assuming a false one, or at times, more than one [14]. The characteristics of the web and the ability of the groomer to maintain continuous contact and access to the victim while ensuring secrecy by deleting messages also make offline perpetrators use the web as a tool in the grooming process [15].

### 1.2. Modus Operandi

Unlike solicitation, which may be a one-time interaction, the process of grooming involves the creation of an intimate relationship that includes five stages: (a) victim selection and the establishment of a confidential relationship as the groomer discloses personal details to gain the victim’s trust; (b) the groomer reinforces the connection, becoming a pivotal figure in the victim’s life; (c) risk assessment, where the predator seeks information about potential obstacles or awareness of the victim’s parents; (d) finding no significant risks, the groomer establishes a relationship characterized by secrecy and exclusivity [10,16]; and (e) sexual stage, which can take various forms and does not necessarily progress to a face-to-face meeting, and discussion of meetings may serve as fantasy enactment [17]. The last stage is marked by the groomer’s initiation of intimate conversations and the exchange of explicit content [10].

Typically, during the grooming process, a perpetrator spends time developing a relationship of trust with the minor being targeted [10]. Perpetrators often take steps to alienate the minors from those they trust, so that the minors are not tempted to report the subsequent exploitation to them. Research suggests that after the requisite level of trust is achieved, perpetrators move to leverage this trust to exploit the minor sexually [11,13]. 

McAlinden [18] pointed out that during the grooming process, perpetrators often use an array of manipulative techniques, including discourse on personal matters, family problems, and social issues. Because the perpetrators are befriending the target, they often engage in a discussion that is free from offensive or suggestive content. At times, sex offenders impersonate young people of the same age as the victim, presenting themselves as having a similar background and common interests [19]. Their goal is to establish trust and eventually normalize sexually harmful behavior. The perpetrator goes about achieving this goal in two steps: first, by preventing the disclosure of the relationship, and second, by laying the groundwork for sexual exploitation.

## 2. Consequences of Sexual Grooming 

Sexual grooming may harm the mental health of adolescent victims and manifest, for example, as depressive symptoms, anxiety, suicidal ideation, and post-traumatic stress [20,21,22,23]. Although the phenomenon of sexual grooming is under-reported and the groomer pressures the victim not to inform the parents about the relationship between them [24,25], it has been found that the parents are an important source of support for the victims and parental support is a significant factor in the rehabilitation of the victims [26]. In addition to post-harm support, parents play an important role in preventing and protecting children from online grooming through mediation and supervision [21,27].

### Parental Control Online

One of the ways to protect children from the negative consequences in the online space is parental mediation [28], which refers to strategies designed to protect children from exposure to online dangers [29,30,31]. Three main strategies are in common use: restrictive mediation, active mediation, and joint use. Restrictive mediation focuses on setting up and enforcing rules for the use of the web concerning the type of content viewed and the duration of the activity. Active mediation provides information, leaves room for discussion of issues, and involves guidance by the parent. Joint use refers to the time parents and children spend together on the Internet watching content or playing, without necessarily discussing the type of content viewed or limiting the length of time spent in the online space. The joint use strategy requires little restriction and supervision because it is suitable for shared activities rather than activities that are primarily personal, such as sending and receiving messages on a smartphone [28]. 

Lack of parental supervision has been found to be associated with risky behaviors in children [32]. It was found that sexual groomers tend to stop communication with adolescents if they believe that there is active or passive parental presence in the victims’ environment [27]. For the purpose of prevention, discussion of the Internet as a sexual space appears to be important for the safe conduct of young people online. When parents perceive a certain phenomenon as threatening to their child, they tend to have an ineffective discussion about it [33,34]. 

As significant figures, parents appear to play an important role in preventive discourse and the treatment of youths after injury by providing guidance and managing the event. A qualitative study by Chiu and Quayle [35] examining adolescents who experienced online grooming with repercussions in the physical space reveals that these adolescents are often unaware of the groomers’ tactics. This lack of awareness significantly impedes their ability to recognize online sexual grooming and avoid becoming victims. Indeed, while previous research has predominantly focused on the direct effects of online grooming on adolescents and prevention strategies, there has been limited exploration of the parental perspective and the broader familial impact. This study seeks to fill this gap by conducting a detailed qualitative analysis of parental experiences and strategies in managing the aftermath of their child’s exposure to online sexual grooming.

## 3. Method

### 3.1. Research Paradigm

The study used a qualitative method that followed the phenomenological approach. It explored the significance and meanings attributed by individuals to phenomena, influencing how they experienced them [36]. By focusing on individuals and the content they chose to present or the meaning they attributed to a phenomenon, the approach made it possible to investigate the phenomenon at the level of consciousness, without bias. This approach permits participants to describe the phenomenon from their subjective point of view, based on their personal experiences, enabling the researcher to comprehend the participant’s first-person experience [37]. In the interpersonal encounter, reality is experienced as it is created, as noted by Spinelli [38]. The present study aimed to gain insight into the world of parents and understand their experience when dealing with adolescents who were victims of online grooming, and the authenticity with which they addressed the issue.

### 3.2. Research Participants 

Fifteen parents (13 mothers) of adolescents who were exposed to online sexual grooming were interviewed (as shown in Table 1). The sample was not intended to be representative of a broader population or to enable generalization. Rather, the intention was to investigate the nature and complexity of individual experiences [39].

The interviewees were recruited through a strategic selection process, utilizing key informant connections as part of a purposeful sampling strategy. Specifically, some participants were identified through personal acquaintances—either directly connected to the researchers or through their broader network. Additionally, other participants were selected following their public disclosures of online grooming events involving their children in various media outlets. This approach ensured a diverse sample of experiences relevant to the study’s focus.

The age of the interviewees at the time of the interview ranged between 37 and 54 years, and they shared experiences of sexual grooming that their boys and girls went through when they were 10–17 years old. The amount of time that had passed since the event ranged from six months to five years. The inclusion criteria were parents whose adolescent children had experienced online grooming. Interviewing continued until saturation was achieved [39].

### 3.3. Procedure

The study was approved by the Institutional Ethics Committee before the interviewees were approached. The semi-structured interviews were conducted by the researchers. 

Following the accepted rules of ethics, in the initial phone conversation and at the beginning of the interview, an explanation was given to participants about the goals of the research and its importance, and they were asked to sign an informed consent form. Participants were also assured that their personal details and those of their children would be kept confidential (by use of pseudonyms and data anonymization through the omission of identifying details). Participants were also told that they could refuse to answer questions and withdraw from the study at any juncture.

The interviews were conducted over Zoom at the convenience of the interviewees and lasted 60–90 min. The interviews were recorded, transcribed in full, and analyzed based on derived themes. 

The semi-structured interviews were conducted using a guide that covered several key themes: the parent–child relationship; dilemmas in handling the grooming event; the grooming event itself; and the consequences of the event on the parent, the child, and the entire family. The guide included questions designed to elicit detailed responses, such as: ‘Could you share any parental dilemmas or doubts regarding the choice of treatment strategy?’, ‘What were the sources of your resilience?’, ‘What actions would you have liked to take but didn’t, and why?’, and ‘In what ways did the event change you or your approach to parenting?’. This approach allowed for comprehensive insights while providing participants the flexibility to express their experiences in depth.

### 3.4. Data Processing and Reliability

Data were processed in three stages. In the first stage, the researchers read the text of each interview as a complete uniform finding from the parent’s point of view, aiming to obtain a holistic view of it. In the second stage, the researchers reread the text and identified units of meaning based on personal perception and interpretation, looking for recurring words, sentences, or ideas that were consolidated into themes [38,40]. In the third stage, the researchers organized, refined, and validated the themes. At the end of this stage, the parents’ points of view regarding their experience of dealing with the sexual grooming of their child emerged.

From the twelfth interview onward, it was observed that the issues being raised were repetitive. Once thematic saturation was reached, as no new information was forthcoming from subsequent interviews, the decision was made to set the sample size at 15 [41]. 

### 3.5. Trustworthiness

To meet the credibility criterion and enhance trustworthiness, the researchers used an audit trail, peer debriefing, and member checking [42,43]. The researchers conducted an in-depth dialogue with the interviewees and asked clarifying questions to achieve a thorough understanding of the phenomenon, reflecting the interviewers’ insights. The authors backed up each idea of the findings with excerpts from the interviews and examined these in light of the findings of other studies in the field. The findings of the present study confirmed those of previous research, which adds to the credibility of the current findings. The research team also discussed the analysis of the findings to avoid bias resulting from personal interpretations. The findings of the present study strengthen those of previous studies, which increases its credibility.

## 4. Findings

**Figure 1:**
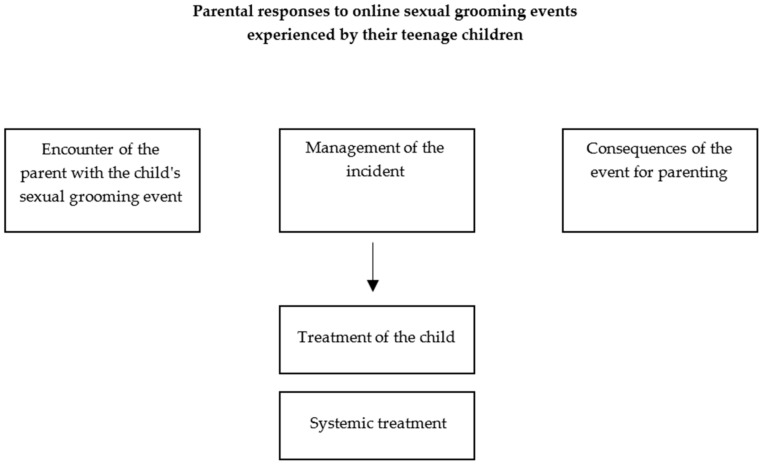
Main themes.

The present study focused on the nature of the parenting experience of parents whose adolescent children were victims of online sexual grooming and on how the participants dealt with the incident. Based on the analysis of the interviews, the research findings were classified into the following main themes (as shown in Figure 1): (a) encounter of the parent with the child’s sexual grooming event; (b) management of the incident, which was divided into two sub-themes: (i) treatment of the child and (ii) systemic treatment; and (c) consequences of the event for parenting.

### 4.1. Encounter with the Child’s Sexual Grooming Event

In all the interviews, the parents stated that the disclosure of the case caused them many negative emotions such as anger, anxiety, and disappointment. The fact that they were not physically or emotionally present at the time their children were attacked by a sex offender made them experience uncertainty. Most of the parents were also surprised to find out that the incident took place in their home, without their knowledge. In none of the mentioned cases of sexual grooming was there a physical meeting between the perpetrator and the victim; however, the cases varied, each one ending at a different stage of harassment. Some cases involved correspondence only; in others, photos were sent; and in one case, there was an intention to meet.

Gili said that she was greatly shocked when she realized that exposure by the police had saved her son from an online relationship with a sex offender. She found out with the help of a police investigator that her son had been corresponding for many weeks with a groomer he had met through an online purchase. This chance meeting led to an exchange of phone numbers and a friendly relationship between the two, continuing into online gaming. “It was scary, stressful. It was a few hours before he got home, and we talked, and I calmed down… those were difficult hours”.

Haim, by contrast, received the information in real time from his daughter, who was distressed by a phone call she received after an online conversation with a groomer. He also related difficult feelings: “As a parent, you think you’re prepared for this situation, but it requires control and confidence. I realized that I have to manage this event. There’s a lot of anger and also irritation, the pressure rises, but I realized that I have to solve it and protect my daughter”. He also shared a feeling of shock: “I was shocked, I saw that there was pressure in the house and that my daughter was scared, but I was also happy because I knew it was under some kind of control”.

Yael described the hard feeling of being greatly disappointed in herself: “I felt terrible. I felt like I wanted to die. How did I not see? How come I was not there in real time? I also felt a kind of disappointment in myself that maybe I didn’t explain to her well enough the dangers that exist on the Internet. I felt threatened. I was so nervous. Why would my daughter even be exposed to such photos? I guard her so closely, and suddenly such information comes out of nowhere. I was really angry! I was less worried because I knew that now I was aware and it was already under control”.

Sarah, who discovered the event by chance, described the moment as one of great shock when she felt the ground give away under her feet: “I took the cell phone and started looking into his WhatsApp, something I used to do every so often, and suddenly I discovered a conversation with this idiot, and I was just shocked, completely shocked, luckily he didn’t delete the correspondence with him, because he kept asking him to delete it… I felt awful. I hear about it on the news, and they explain all the time at school, and it happened right here under my nose. In my house, in my child’s room. Like, how did it happen!!! And I was very angry with myself that I didn’t take notice on the one hand, and on the other, it’s lucky that we caught on to it in time”.

All the parents who were interviewed appear to have been surprised and horrified when they were exposed to their children’s harmful incident. They felt guilty for not being able to protect their children and tried to regain a sense of control. They all stated that the very knowledge made them feel in control of the situation and that the power was back in their hands.

### 4.2. Managing the Event

All the interviewees took an active part in the management of the event. The way the incident was handled can be regarded at two levels: the treatment of the child who sustained the injury and the systemic treatment.

 *a.* *Treatment of the child*

The interviewed parents described their reactions to their child after the harm. The reactions, resulting from helplessness, ranged from anger to empathy and even concealment from the other parent. 

Shira, for example, discovered that her daughter thought she was corresponding with a peer of her own age. She shared the feelings of anger and shock that were expressed in her reaction toward her daughter: “Wow, I panicked. I was really on edge!!! I called her right away, I even got angry at her, I don’t know why, but I yelled at her to immediately bring me her phone”. Later, she shared the feeling of embarrassment that prevented her from telling her husband because their daughter had corresponded in a sexually explicit manner.

It seems that how the relationship was discovered and the stage the child and the groomer had reached (sexual or only friendly conversation) affected the way the parents reacted. For example, Deborah, whose son became suspicious and shared with her his communication with the groomer, described how she complimented her son for being alert: “I told him I was proud of him for his instincts and I felt relieved that he shared the information with me”.

As part of handling the incident, most parents chose to provide an emotional response to the child, which included psychotherapy or other emotional treatment. Some parents, however, preferred to carry out the therapy themselves. Yael, for example, related: “I saw my daughter so fragile, I suggested that she go to a professional so that she could talk and unload it all. She immediately agreed. I felt that she wanted it very much and was open to it. She went to several therapy sessions”. One mother shared that her son needed psychiatric treatment because of the great distress and emotional anxiety caused by the crisis he experienced: “I took him to a psychiatrist three times. He acted in a crazy way, he wanted to jump off the balcony”. Others chose not to send their children to emotional therapy but provided emotional and social support, constantly paying attention to the emotional state of the adolescent.

Some parents reported that the event did not leave a deep mark on their children and claimed that it would pass as if it had not happened, especially in cases where communication was cut off quickly.

 *b.* *Systemic treatment*

The systemic treatment included mainly the school and the police. Although the school is supposed to be a partner in supporting the children, most parents did not trust it, indicating either a lack of trust in the ability of the education system to handle such events or that they were not satisfied with the way the school had handled the situation. Mia, for example, said: “We informed the school, the homeroom teacher, and the counselor. There were several conversations within the school setting, but I didn’t count on it”.

Other parents also stated that they informed the school and that the educational staff did not stand by their children as they had expected, or demonstrated helplessness, not knowing how to handle the events. Other parents, however, claimed that because the events did not take place within the school, they did not think it was necessary to involve the school.

In both cases, according to the parents’ reports, the treatment provided by the school, if any, appeared to have been limited, and the parents were ambivalent about its effectiveness. At the same time, all the parents wanted increased awareness and information on the part of the school and mentioned the importance of the youths learning about the risks.

Alongside turning to the school, which was not always considered a solution, most parents chose to immediately report the incident to the police. Emma, for example, said: “I reported it to the police immediately. I didn’t wait. I didn’t want to give him [groomer] a chance to send a picture again or call at night”.

Haim said that before contacting the police, he preferred to obtain the details of the groomer: “We decided to pretend to be my daughter and correspond with him on her behalf. I tried to reveal as much information as possible about how old he is, where he lives, and at the same time, I started looking for him on Facebook. I already have his phone number, I also have the photo, and what I did was to try to find him on Facebook… I was able to find him relatively quickly. I even got to his address, the exact residential address, and when I had everything, then the fear eased. I wrote to him: ‘This is her father. I have pictures of everything and I’m calling the police.’ At that moment he blocked me…”

Most parents reported satisfaction with the efficiency of the police handling their case, except for two parents who were disappointed with the police and the prosecutor’s office, as illustrated by the following statement: “The handling was very bad because there was no handling. After I filed the complaint, a few months later, the case was closed, and why? Because he left the country and ran away. That is, if a person does something and then flies abroad, he’s exempt?”

### 4.3. The Consequences of the Event for Parenting

Most parents felt that the way they handled the event helped in several ways. First, it allowed them to build trust between themselves and their children. Second, it allowed them to reassure their children that they were a source of support and resilience. Haim said: “She trusts me. She said a sentence that, for me, is worth everything… She said: I didn’t feel anything because I knew my father was taking care of it. I mean, she knows I can be trusted and that’s what’s important.” Conversely, Benjamin revealed that after the event, a distance materialized between him and his son, which was the result, among others, of the father’s lack of tools to deal with his son’s emotional state: “He started having anxiety attacks that I didn’t know how to recognize because I’m not in this field… and then I really felt powerless… during anxiety attacks I felt that he sealed himself off, that I couldn’t talk to him, and actually it was exactly like that because you suddenly know you have a child at home who doesn’t want to live and you can’t talk to him, you can’t convince him that everything is fine because he’s sure that everything isn’t right and everything is falling apart.” The child’s perception of the parent’s treatment appears to have had a decisive effect on the nature of the relationship after the incident.

The interviewees also mentioned the consequences of the case for their parenting. They shared their dilemmas about their children’s need for independence and the anxiety involved in granting them this independence. The parents said that since the event, they have been asking more questions and taking more interest in their children’s online social life. One of the mothers described it thus: “This independence comes at a price. I’m more worried. More anxious. It’s a kind of dance between giving him independence and watching over him and being there.”

Some parents discussed their parenting of younger siblings and described the stricter parental supervision measures they instituted. One parent described this as “Keeping your finger more intensely on the pulse. For example, my little son has exactly the same computer the older one has, so I don’t check every two weeks but every day. Who are you talking to? I talk to whoever is talking, I’m always on it. There’s nothing else to do.” Some of the parents tried to monitor what was happening in the online space both by exercising control within the clear boundaries that characterize restrictive mediation and by active mediation that takes place through dialogue and understanding. The interviews also reflected the parental understanding that it is important to explain and communicate with their children to ensure protection in the online space. Some of the parents said that they checked how their children conducted themselves on their smartphones and read their conversations. For example, Nili said: “I have no interest in privacy and all this nonsense, what’s privacy? It’s a 13-year-old child. He’s a child, and he’s my responsibility, and my responsibility is to check what’s going on. When he’s 18 and when he gets married, let him do whatever he wants on his own responsibility.” In the same matter, Yael related: “I don’t pry and read everything. Absolutely not. I just skim and try to leave room for her privacy. Only if I see something unusual will I confront her. In the end, they prefer to share with their friends and not with us parents.”

The event appears to have caused the parents to be more aware and vigilant, and it intensified their anxiety and supervision of the children’s behavior on the Internet and social media.

## 5. Discussion

The present study examined the experiences of parents of adolescent victims of online sexual grooming, focusing on the parents’ attitude toward protection and supervision in the online space, the emotional aspects of the parents’ coping, and how they managed the incident. The study also examined the effect of the event on the parents and discussed whether it changed them or their parenting.

The findings show that the parents were greatly surprised by the incident that had occurred in their family and unbelieving that such a thing could happen in their home. They were also surprised to find out that their children were exposed to sexual content online. Previous studies have found that parents did not have accurate information about their children’s online activities [44]. Only 40% of the parents claimed that they always knew what their children were doing online, about 52% said that they sometimes knew what their children were doing, and about 5% claimed that they did not know at all what their children were doing online. It was also found that 20% of adolescents reported that their parents had no idea what they were doing online [45]. These findings are consistent with those of the current study, which reflect the parents’ astonishment at the incident.

Although sex education is an important aspect of adolescents’ lives, parents often tend to ignore it, and most of them have difficulty communicating about sexual issues with their children [46]. Studies further indicate that discourse about sexuality causes dilemmas and difficulties for parents [33,47]. Furthermore, the results of the present study indicate that although participants’ conversations with their children addressed online dangers, they did not touch upon the topic of sexuality. This finding is consistent with research showing that parents have difficulty talking to their children about sexual issues, fearing that the subject is too sensitive for their children and may not be suitable for their age or understanding [48].

Studies show that there is a connection between the way a parent reacts to an event and the child’s experience of the event and that the experience of coping together with difficulties in the lives of the adolescent and the parent allows parents to become models for coping and adaptation in different situations in the child’s life [49,50]. In the present study, it was found that parents who showed confidence in their children gave them a sense of protection and security during the sexual grooming incident. 

It may be argued that life crises are stressors, and their frequency and the way of coping with them are related to difficulties in emotional control. They harm and can degrade relationships between parents and their children [51]. Therefore, parents have emotional difficulty handling a discussion about sexuality with their children, even if this is essential for ensuring the children’s safety and even if a real risk may result from their not knowing enough about the subject. 

In the present study, it was found that all the parents interviewed reported having done everything they could to properly manage the incident and protect their children while also making contact with other parties and seeking their help in handling the incident. The parents’ actions consisted, among others, of contacting the police and officials within the education system, such as the homeroom teacher and the guidance counselor. Management of the event varied from person to person, and different strategies were used to deal with the stressful situations. 

Two main strategies are proposed for coping with stressful situations: problem-focused and emotion-focused. Problem-focused coping includes taking the initiative, being active, and dealing with the source of the stress. Emotion-focused coping seeks to reduce the emotional stress in the wake of the event [52]. The findings of the present study show that when managing the event, the interviewees acted operatively: it was of great importance for them to minimize damage, establish contact with supportive actors who could protect their children, and shield the adolescent from the perpetrator. The parents also reported that as part of dealing with the incident, they sought emotional support for their children, and approached the school counselor and other therapists. Research on coping with experiences of sexual harassment reveals that there is no single strategy that is most appropriate for managing a sexual crisis. Some support problem-focused coping arguing that it is more effective for the victim [53]. Others, however, point out that the problem-focused approach may increase the risk of harm to the victim and lead to difficulty coping with the crisis [54].

The present study examined coping from the perspective of the parents of the adolescents rather than from that of the adolescents themselves, relying on the parents’ reports about the management strategy and handling of the case. Participating parents reported a desire to manage the incident and handle the problem in a targeted manner that did not emphasize the emotional implications for the adolescent. The parents claimed that they did so out of a desire to provide real protection for their children and to complete the handling of the incident as quickly as possible. According to previous studies, there are substantial gaps between what parents know about their children and what the children do and feel. Most parents expressed a desire to monitor what happens online [55].

The literature indicates that when children experience an emotional crisis and the parents handle the situation, it has a lasting effect on the parents’ behavior and their subsequent functioning. This is primarily due to the fact that the responsibility for tending to the child’s well-being ultimately remains with the parents [56]. The findings of the present study show that the parents experienced a crisis following the sexual harassment of their children. They had to act as a safe haven for their children even though they themselves faced a great emotional upheaval. It can be seen from the parental behavior that they acted to protect their child and felt in control from the moment they learned about the event and addressed it. Studies have found that restoring a sense of control is important for functioning in trauma situations. Research suggests that when control is taken away, regaining it should be encouraged because it allows continued functioning [57].

In the present study, the parents reported that after the event, they increased the supervision and protection of the younger siblings in the family. This finding is consistent with the results of another study that examined changes in parenting styles and Internet use and reported that parents changed their level of control over Internet use and supervised the younger siblings relatively more than their older ones [58]. A key understanding related to parent perceptions is that parents and family members have greatly diverging views and ideas about what is best for their children [59]. This finding is reinforced by other studies of online sexual abuse, which indicate that educational counselors do not know how to deal with incidents of online abuse or do not recognize them as significant events [60,61]. This may be the reason why parents reported that they were the most appropriate person to manage the incidents of sexual abuse of their children [62].

It appears, therefore, that in cases of online sexual grooming, the management of the case and the reporting, together with protecting the wellbeing of the victim, falls on the shoulders of the parents, who adapt their response to the needs of the victims, adjusting their parenting accordingly, based on how they perceive the factors that can help. Increasing awareness in the education system regarding prevention and the creation of post-event intervention plans, together with understanding the need for joint systemic care after harmful events could make it easier for both the coping parents and the harmed adolescent.

## 6. Practical Implementation

The findings of the present study suggest that there is room for improvement in the cooperation with police and educational authorities. Many of the interviewees suggested that the police were an effective factor in the process whereas the educational staff did not know how to cope with the harmful event. 

Because of the central roles played by the police and educational system, it is important to develop training and educational programs targeted at law enforcement (including the police), gatekeepers (including those working in the educational system, caregivers, and parents), and children, providing them with the knowledge, skills, strategies, and tools needed to identify and confront child sexual exploitation and abuse (CSEA) generally, and sexual grooming specifically. 

The importance of training and bespoke educational programs is confirmed by several international organizations (including UNICEF, the World Health Organization, and the European Union) and NGOs (such as the WeProtect Alliance) (EU: Directive 200/92/EU of the European Parliament and of the Council of 13 December 2011) combating the sexual abuse and sexual exploitation of children and child pornography. The creation of a cross-border infrastructure involving the public and private sectors is currently being deliberated upon within the European Union and is part of reform put forward by the European Commission (European Commission’s Proposal for a Regulation of the European Parliament and of the Council COM (2022) 209 final (Brussels, 11 May 2022)) [63]. In partnership with UNICEF and with the financial support of the European Union, the WeProtect Alliance developed a multifaceted strategy, called the Model National Response (Model, for short), aimed at providing nations with the tools needed to (a) flag and mitigate the risks of child online sexual exploitation and (b) respond effectively to such incidents when they arise by putting in place a complete national response. Acknowledging that such a response requires a broad set of national capabilities, the WeProtect Alliance calls for a comprehensive systemic strategy along six fronts: improvements in policy, legislation, and governance; changes in the criminal justice system; improvements in victim support and empowerment; and launching of a coordinated public–private dialogue to find solutions to the problem. The Model also calls for the introduction and development of national educational programs (including age-appropriate, inclusive, and accessible content) to raise children’s and gatekeepers’ awareness of CSEA (including parents, caregivers, guardians, teachers, social care workers, health practitioners, etc.).

Alongside the call for the development of targeted training and educational programs, the present paper calls for the development of trauma-informed and victim-focused training for law enforcement personnel and those accompanying children and their families after such incidents. Inspired by the recommendations in the Model, this paper suggests the development of child-friendly and victim-centered protocols as part of a national support strategy in coordination with victim support services, and the provision of specialist training for personnel working for the criminal justice system (including law enforcement, the judiciary, and the prosecutorial service) and other public service personnel who accompany the children and their families in the aftermath of the event, including those working in the school system.

By ensuring the provision of such training, the individuals handling the investigation, prosecution, and enforcement processes will have the necessary expertise to handle these cases and the skills needed to support, guide, and counsel the victims and their families, referring them to specialist services when necessary. The same is true for those working in the school system. 

The efforts to support, guide, and counsel the victims and their families should be part of a broader strategy aimed at facilitating victim recovery and supporting their caregivers, including their families. With this in mind, this paper suggests the provision of specialist training to those working in the educational system, including teachers and school staff, so that they can assist victims and refer them to specialists while providing the student body as a whole with the tools to support victims if information about the abuse is shared with them.

## 7. Limitations and Directions for Future Studies

The findings of this qualitative research cannot be generalized to represent the views of all parents whose adolescent children have experienced online grooming. Nevertheless, the study examined the perceptions of participating parents, uncovering new and potentially fruitful areas for future research. 

The interviews were conducted over Zoom, which may have influenced the openness and closeness between the interviewees and the interviewers. At the same time, conducting the interview remotely made it possible to reach parents from many regions of the country who would not necessarily travel to physically participate in the interviews.

A total of 13 women and 2 men participated in the study. Men and women respond differently to stressful life events, exhibiting distinct patterns of psychological distress and emotional coping styles [64,65]; therefore, it is important to conduct a study in which fathers have a more significant voice. The researchers had great difficulty locating interviewees because of the sensitivity of the topic and the fear of exposure. Further research should examine the differences between fathers and mothers in the way they experience and behave in the face of the online grooming phenomenon. Finally, it is important to interview the victims themselves, the adolescents, about how they experienced the guidance of their parents in the course of their injury. 

## Figures and Tables

**Table 1:** Demographic characteristics of the interviewees.

	Nick Name	Gender	Age	Marital Status	Age of the Adolescents at the Time of the Event	Child Gender	Time Since Abuse
1	Deborah	Woman	54	Separated	11	Boy	2 years
2	Haim	Man	41	Married	10.5	Girl	1/2 year
3	Shira	Woman	54	Married	14	Girl	1 year
4	Yael	Woman	45	Married	11	Girl	1 year
5	Sarah	Woman	44	Married	12	Boy	5 years
6	Benjamin	Man	48	Married	12	Boy	5 years
7	Mia	Woman	47	Married	12	Girl	2 years
8	Sophie	Woman	53	Divorced	13	Girl	2 years
9	Natalie	Woman	47	Married	14	Girl	3 years
10	Zoe	Woman	40	Married	11	Girl	2 years
11	Sofia	Woman	52	Married	15	Girl	2 years
12	Emma	Woman	45	Married	12.5	Girl	2.5 years
13	Gili	Woman	42	Married	13	Boy	1 year
14	Ella	Woman	45	Married	12	Girl	3.5 years
15	Nili	Woman	43	Married	11	Boy	2 years

## Data Availability

The datasets used in this article are not publicly available due to confidentiality constraints and their presentation in Hebrew. For inquiries regarding access to the datasets, please contact M.D.-C.
